# Expanding the Detection of Traversable Area with RealSense for the Visually Impaired

**DOI:** 10.3390/s16111954

**Published:** 2016-11-21

**Authors:** Kailun Yang, Kaiwei Wang, Weijian Hu, Jian Bai

**Affiliations:** College of Optical Science and Engineering, Zhejiang University, Hangzhou 310027, China; elnino@zju.edu.cn (K.Y.); huweijian@zju.edu.cn (W.H.); bai@zju.edu.cn (J.B.)

**Keywords:** RGB-D sensor, RealSense, visually impaired people, traversable area detection

## Abstract

The introduction of RGB-Depth (RGB-D) sensors into the visually impaired people (VIP)-assisting area has stirred great interest of many researchers. However, the detection range of RGB-D sensors is limited by narrow depth field angle and sparse depth map in the distance, which hampers broader and longer traversability awareness. This paper proposes an effective approach to expand the detection of traversable area based on a RGB-D sensor, the Intel RealSense R200, which is compatible with both indoor and outdoor environments. The depth image of RealSense is enhanced with IR image large-scale matching and RGB image-guided filtering. Traversable area is obtained with RANdom SAmple Consensus (RANSAC) segmentation and surface normal vector estimation, preliminarily. A seeded growing region algorithm, combining the depth image and RGB image, enlarges the preliminary traversable area greatly. This is critical not only for avoiding close obstacles, but also for allowing superior path planning on navigation. The proposed approach has been tested on a score of indoor and outdoor scenarios. Moreover, the approach has been integrated into an assistance system, which consists of a wearable prototype and an audio interface. Furthermore, the presented approach has been proved to be useful and reliable by a field test with eight visually impaired volunteers.

## 1. Introduction

According to the World Health Organization, 285 million people were estimated to be visually impaired and 39 million of them are blind around the world in 2014 [1]. It is very difficult for visually impaired people (VIP) to find their way through obstacles and wander in real-world scenarios. Recently, RGB-Depth (RGB-D) sensors revolutionized the research field of VIP aiding because of their versatility, portability, and cost-effectiveness. Compared with traditional assistive tools, such as a white cane, RGB-D sensors provide a great deal of information to the VIP. Typical RGB-D sensors, including light-coding sensors, time-of-flight sensors (ToF camera), and stereo cameras are able to acquire color information and perceive the environment in three dimensions at video frame rates. These depth-sensing technologies already have their mature commercial products, but each type of them has its own set of limits and requires certain working environments to perform well, which brings not only new opportunities but also challenges to overcome.

Light-coding sensors, such as PrimeSense [2] (developed by PrimeSense based in Tel Aviv, Israel), Kinect [3] (developed by Microsoft based in Redmond, WA, USA), Xtion Pro [4] (developed by Asus based in Taipei, Taiwan), MV4D [5] (developed by Mantis Vision based in Petach Tikva, Israel), and the Structure Sensor [6] (developed by Occipital based in San Francisco, CA, USA) project near-IR laser speckles to code the scene. Since the distortion of the speckles depends on the depth of objects, an IR CMOS image sensor captures the distorted speckles and a depth map is generated through triangulating algorithms. However, they fail to return an efficient depth map in sunny environments because projected speckles are submerged by sunlight. As a result, approaches for VIP with light-coding sensors are just proof-of-concepts or only feasible in indoor environments [7,8,9,10,11,12,13,14,15].

ToF cameras, such as CamCube [16] (developed by PMD Technologies based in Siegen, Germany), DepthSense [17] (developed by SoftKinetic based in Brussels, Belgium), and SwissRanger (developed by Heptagon based in Singapore) [18] resolve distance based on the known speed of light, measuring the precise time of a light signal flight between the camera and the subject independently for each pixel of the image sensor. However, they are susceptible to ambient light. As a result, ToF camera-based approaches for VIP show poor performance in outdoor environments [19,20,21].

Stereo cameras, such as the Bumblebee [22] (developed by PointGrey based in Richmond, BC, Canada), ZED [23] (developed by Stereolabs based in San Francisco, USA), and DUO [24] (developed by DUO3D based in Henderson, NV, USA) estimates the depth map through stereo matching of images from two or more lenses. Points on one image are correlated to another image and depth is calculated via shift between a point on one image and another image. Stereo matching is a passive and texture-dependent process. As a result, stereo cameras return sparse depth images in textureless indoor scenes, such as a blank wall. This explains why solutions for VIP with stereo camera focus mainly on highly-textured outdoor environments [25,26,27,28].

**Figure 1:**
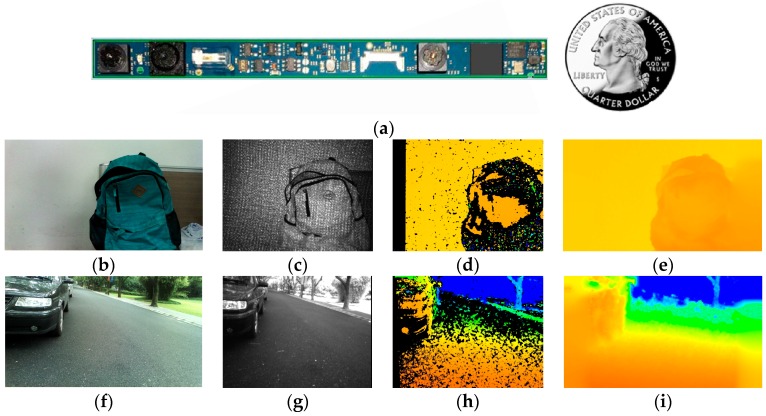
(**a**) The RealSense R200; (**b**,**f**) color image captured by the RealSense R200; (**c**,**g**) IR image captured by the right IR camera of the RealSense R200; (**d**,**h**) the original depth image from the RealSense R200; and (**e**,**i**) the guided filtered depth image acquired in our work.

The RealSense R200 (developed by Intel based in Santa Clara, CA, USA) uses a combination of active projecting and passive stereo matching [29]. IR laser projector projects static non-visible near-IR patterns on the scene, which is then acquired by the left and right IR cameras. The image processor generates a depth map through an embedded stereo-matching algorithm. In textureless indoor environments, the projected patterns enrich textures. As shown in Figure 1b,c, the texture-less white wall has been projected with many near-IR patterns which are beneficial for stereo matching to generate depth information. In sunny outdoor environments, although projected patterns are submerged by sunlight, the near-IR component of sunlight shines on the scene to form well-textured IR images as shown in Figure 1g. With the contribution of abundant textures to robust stereo matching, the combination allows the RealSense R200 to work under indoor and outdoor circumstances, delivering depth images though it has many noise sources, mismatched pixels, and black holes. In addition, it is possible to attain denser depth maps pending new algorithms. Illustrated in Figure 1, the RealSense R200 is quite suitable for navigational assistance thanks not only to its environment adaptability, but also its small size.

**Figure 2:**
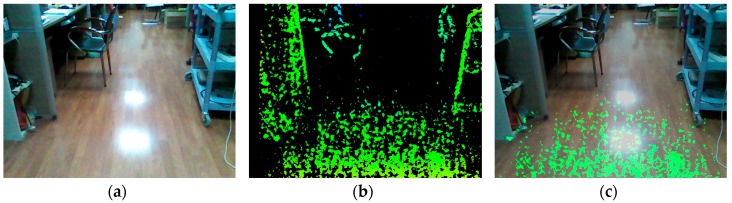
(**a**) Color image captured by the RealSense R200; (**b**) the original depth image captured by the RealSense R200; (**c**) traversable area detection with original depth image of the RealSense R200, which is limited to short range.

**Figure 3:**
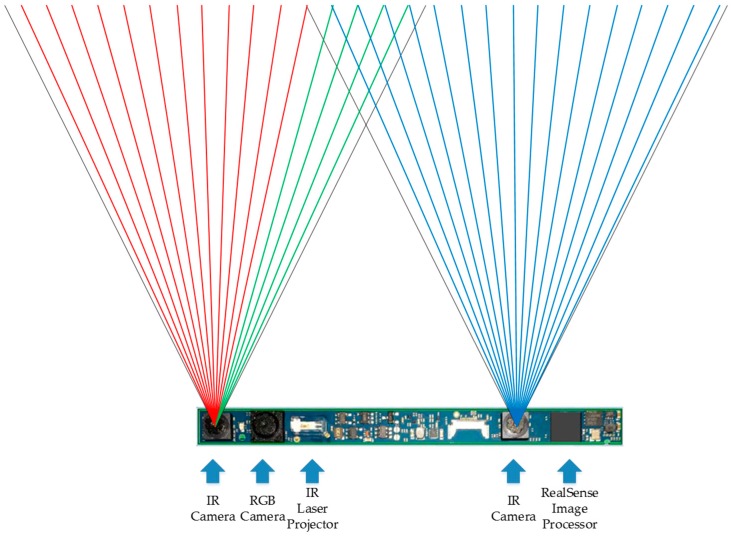
Horizontal field angle of IR cameras.

However, the depth range of the RGB-D sensor is generally short. For the light-coding sensor, the speckles in the distance are too dark to be sensed. For the ToF camera, light signals are overwhelmed by ambient light in the distance. For stereo-cameras, since depth error increases with the increase of the depth value, stereo-cameras are prone to be unreliable in the distance [30]. For the RealSense R200, on the one hand, since the power of IR laser projector is limited, if the coded object is in the distance, the speckles are too dark and sparse to enhance stereo matching. On the other hand, depth information in the distance is much less accurate than that in the normal working distance ranging from 650–2100 mm [31]. As shown in Figure 2, the original depth image is sparse a few meters away. In addition, the depth field angle of RGB-D sensor is generally small. For the RealSense R200, the horizontal field angle of IR camera is 59°. As we know, the depth image is generated through stereo matching from overlapping field angles of two IR cameras. Illustrated in Figure 3, though red and green light are within the horizontal field angle of the left IR camera, only green light is within the overlapping field angle of two IR cameras. Thus, the efficient depth horizontal field angle is smaller than 59°, which is the horizontal field angle of a single IR camera. Consequently, as depicted in Figure 2, both the distance and the angle range of the ground plane detection with the original depth image are small, which hampers longer and broader traversable area awareness for VIP.

In this paper, an effective approach to expand the traversable area detection is proposed. Since the original depth image is poor and sparse, two IR images are large-scale matched to generate a dense depth image. Additionally, the quality of the depth image is enhanced with the RGB image-guided filtering, which is comprised of functions, such as de-noising, hole-filling, and can estimate the depth map from the perspective of the RGB camera, whose horizontal field angle is wider than the depth camera. The preliminary traversable area is obtained with RANdom SAmple Consensus (RANSAC) segmentation [32]. In addition to the RealSense R200, an attitude sensor, InvenSense MPU6050 [33], is employed to adjust the point cloud from the camera coordinate system to the world coordinate system. This helps to eliminate sample errors in preliminary traversable area detection. Through estimating surface normal vectors of depth image patches, salient parts are removed from preliminary detection results. The highlighted process of the traversable area detection is to extend preliminary results to broader and longer ranges, which fully combines depth and color images. On the one hand, short-range depth information is enhanced with long-range RGB information. On the other hand, depth information adds a dimension of restrictions to the expansion stage based on seeded region growing algorithm [34]. The approach proposed in this paper is integrated with a wearable prototype, containing a bone-conduction headphone, which provides a non-semantic stereophonic interface. Different from most navigational assistance approaches, which are not tested by VIP, eight visually impaired volunteers, three in whom are suffering from total blindness, have tried out our approach. 

This paper is organized as follows: in Section 2, related work that has addressed both traversable area detection and expansion are reviewed; in Section 3, the presented approach is elaborated in detail; in Section 4, extensive tests on indoor and outdoor scenarios demonstrate its effectiveness and robustness; in Section 5, the approach is validated by the user study, effected by real VIP; and in Section 6, relevant conclusions are drawn and outlooks to future work are depicted.

## 2. Related Work

In the literature, a lot of approaches have been proposed with respect to ground plane segmentation, access section detection, and traversable area awareness with RGB-D sensors. 

In some approaches, ground plane segmentation is the first step of obstacle detection, which aims to separate feasible ground area from hazardous obstacles. Wang adopted meanshift segmentation to separate obstacles based on the depth image from a Kinect, in which planes are regarded as feasible areas if two conditions are met: the angle between the normal vector of the fitting plane and vertical direction of the camera coordinate system is less than a threshold; and the average distance and the standard deviation of all 3D points to the fitting plane are less than thresholds [35]. Although the approach achieved good robustness under certain environment, the approach relies a lot on thresholds and assumptions. Cheng put forward an algorithm to detect ground with a Kinect based on seeded region growing [15]. Instead of focusing on growing thresholds, edges of the depth image and boundaries of the region are adequately considered. However, the algorithm is unduly dependent on the depth image, and the seed pixels are elected according to a random number, causing fluctuations between frames, which is intolerable for assisting because unstable results would confuse VIP. Rodríguez simply estimated outdoor ground plane based on RANSAC plus filtering techniques, and used a polar grid representation to account for the potential obstacles [25]. The approach is one of the few which have involved real VIP participation. However, the approach yields a ground plane detection error in more than ten percent of the frames, which is resolvable in our work.

In some approaches, the problem of navigable ground detection is addressed in conjunction with localization tasks. Perez-Yus used the RANSAC algorithm to segment planes in human-made indoor scenarios pending dense 3D point clouds. The approach is able to extract not only the ground but also ascending or descending stairs, and to determine the position and orientation of the user with visual odometry [36]. Lee also incorporated visual odometry and feature-based metri-topological simultaneous localization and mapping (SLAM) [37] to perform traversability analysis [26,38]. The navigation system extracts ground plane to reduce drift imposed by the head-mounted RGB-D sensor and the paper demonstrated that the traversability map works more robustly with a light-coding sensor than with a stereo pair in low-textured environments. As for another indoor localization application, Sánchez detected floor and navigable areas to efficiently reduce the search space and thereby yielded real-time performance of both place recognition and tracking [39].

In some approaches, surface normal vectors on the depth map have been used to determine the accessible section. Koester detected the accessible section by calculating the gradients and estimating surface normal vector directions of real-world scene patches [40]. The approach allows for a fast and effective accessible section detection, even in crowded scenes. However, it prevents practical application for user studies with the overreliance on the quality of 3D reconstruction process and adherence to constraints such as the area directly in front of the user is accessible. Bellone defined a novel descriptor to measure the unevenness of a local surface based on the estimation of normal vectors [41]. The index gives an enhanced description of the traversable area which takes into account both the inclination and roughness of the local surface. It is possible to perform obstacle avoidance and terrain traversability assessments simultaneously. However, the descriptor computation is complex and also relies on the sensor to generate dense 3D point clouds. Chessa derived the normal vectors to estimate surface orientation for collision avoidance and scene interpretation [42]. The framework uses a disparity map as a powerful cue to validate the computation from optic flow, which suffers from the drawback of being sensitive to errors in the estimates of optical flow.

In some approaches, range extension are concerned to tackle the limitations imposed by RGB-D sensors. Muller presented a self-supervised learning process to accurately classify long-range terrain as traversable or not [43]. It continuously receives images, generates supervisory labels, trains a classifier, and classifies the long-range portion of the images, which complete one full cycle every half second. Although the system classifies the traversable area of the image up to the horizon, the feature extraction requires large, distant image patches within fifteen meters, limiting the utility in general applications with commercial RGB-D sensors, which ranges mush closer. Reina proposed a self-learning framework to automatically train a ground classifier with multi-baseline stereovision [44]. Two distinct classifiers include one based on geometric data, which detects the broad class of ground, and one based on color data, which further segments ground into subclasses. The approach makes predictions based on past observations, and the only underlying assumption is that the sensor is initialized from an area free of obstacles, which is typically violated in applications of VIP assisting. Milella features a radar-stereo system to address terrain traversability assessment in the context of outdoor navigation [45,46]. The combination produces reliable results in the short range and trains a classifier operating on distant scenes. Damen also presented an unsupervised approach towards automatic video-based guidance in miniature and in fully-wearable form [47]. These self-learning strategies make feasible navigation in long-range and long-duration applications, but they ignore the fact that most traversable pixels or image patches are connected parts rather than detached, which is fully considered in our approach, and also supports an expanded range of detection. Aladrén combines depth information with image intensities, robustly expands the range-based indoor floor segmentation [9]. The overall diagram of the method composes complex processes, running at approximately 0.3 frames per second, which fails to assist VIP at normal walking speed.

Although plenty of related works have been done to analyze traversable area with RGB-D sensors, most of them are overly dependent on the depth image or cause intolerable side effects in navigational assistance for VIP. Compared with these works, the main advantages of our approach can be summarized as follows:

- The 3D point cloud generated from the RealSense R200 is adjusted from the camera coordinate system to the world coordinate system with a measured sensor attitude angle, such that the sample errors are decreased to a great extent and the preliminary plane is segmented correctly.
- The seeded region, growing adequately, considers the traversable area as connected parts, and expands the preliminary segmentation result to broader and longer ranges with RGB information.
- The seeded region growing starts with preliminarily-segmented pixels other than according to the random number, thus the expansion is inherently stable between frames, which means the output will not fluctuate and confuse VIP. The seeded region growing is not reliant on a single threshold, and edges of the RGB image and depth differences are also considered to restrict growing into non-traversable area.
- The approach does not require the depth image from sensor to be accurate or dense in long-range area, thus most consumer RGB-D sensors meet the requirements of the algorithm.
- The sensor outputs efficient IR image pairs under both indoor and outdoor circumstances, ensuring practical usability of the approach.

## 3. Approach

**Figure 4:**
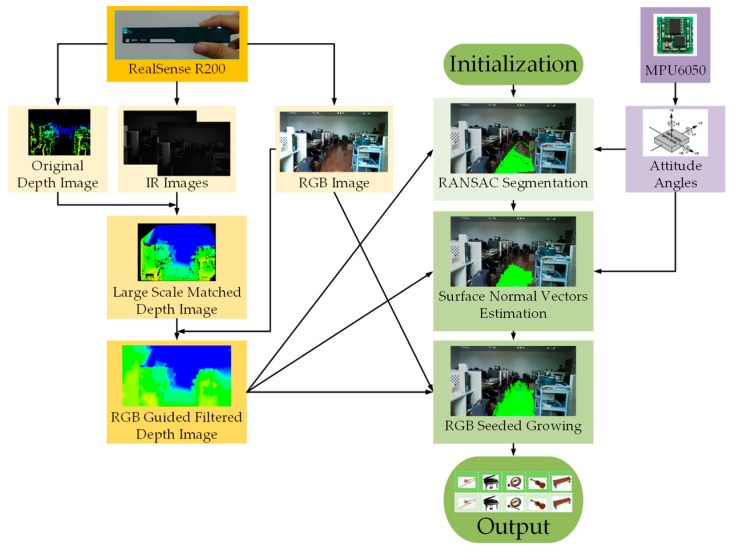
The flowchart of the approach.

In this section, the approach to expand traversable area detection with the RealSense sensor is elaborated in detail. The flow chart of the approach is show in Figure 4. The approach is described in terms of depth image enhancement, preliminary ground segmentation, and seeded region expansion, accordingly.

### 3.1. Depth Image Enhancement

The original depth image from the RealSense R200 is sparse and there are many holes, noises, and mismatched pixels. Besides, the embedded stereo-matching algorithm in the processor is fixed, which is unable to be altered. The embedded algorithm is based on local correspondences, and parameters are fixed with the algorithm, such as the texture threshold and uniqueness ratio, limiting the original depth map to be sparse. Typical original depth images are shown in Figure 1d,h. Comparatively, IR images from the RealSense are large-scale matched in our work.

**Figure 5:**
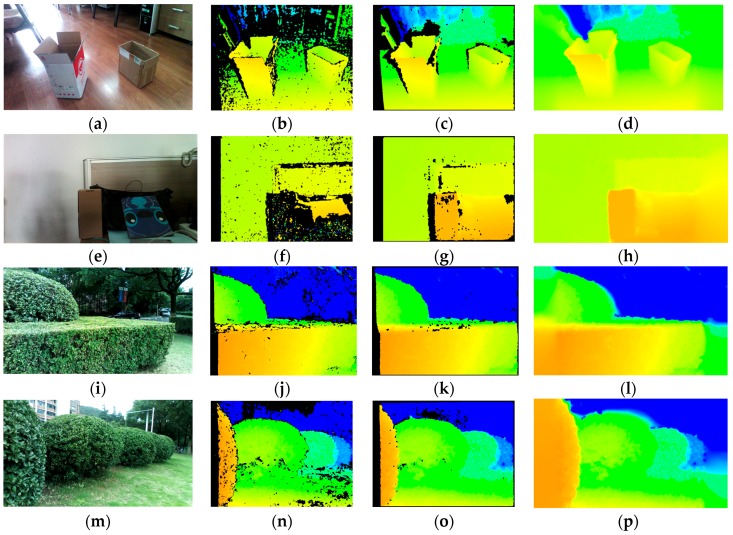
Comparison of depth maps under both indoor and outdoor environments. (**a**,**e**,**i**,**m**) Color images captured by the RealSense sensor; (**b**,**f**,**j**,**n**) original depth image from the RealSense sensor; (**c**,**g**,**k**,**o**) large-scale matched depth image; and (**d**,**h**,**l**,**p**) guided-filter depth image.

To yield a dense depth map with calibrated IR images, original efficient depth pixels are included in the implementation of efficient large-scale stereo matching algorithm [48]. Support pixels are denoted as pixels which can be robustly matched due to their textures and uniqueness. Sobel masks with fixed size of 3 × 3 pixels and a large disparity search range are used to perform stereo matching and obtain support pixels. As Sobel filter responses are good, but still insufficient, for stereo matching, original depth image pixels are added to the support pixels. In addition, a multi-block-matching principle [49] is employed to obtain more robust and sufficient support matches from real-world textures. Given the resolution of IR images is 628 × 468, the best block sizes found with IR pairs are 41 × 1, 1 × 41, 9 × 9, and 3 × 3. Then, the approach estimates the depth map by forming triangulation on a set of support pixels and interpolating disparities. As shown in Figure 5, the large-scale matched depth image is much denser than the original depth map, especially in less-textured scenarios, even though these original depth images are the denser ones acquired with the sensor.

However, there are still many holes and noises in the large-scale depth image. Moreover, the horizontal field view of the depth image is narrow, which hampers broad navigation. In order to take advantage of available color images acquired with the RealSense R200 instead of filling invalid regions in a visually plausible way using only depth information, we incorporate a color image and apply the guided filter [50] to refine and estimate the depth of unknown areas. In this work, we implement a RGB guided filter within the interface of enhanced photography algorithms [51] to improve the depth image, which is to fill holes, de-noise and, foremost, estimate the depth map from the field view of the RGB camera. The color image, depth image, and calibration data are input to the post-process, within which the original depth image is replaced by a large-scale matched depth image. Firstly, depth information from the perspective of one IR camera is projected onto the RGB image with both IR cameras and the RGB camera calibration parameters. In this process, depth values are extracted from the large-scale matched depth image instead of original depth image. Secondly, a color term is introduced so that the weighting function in the guided filter is able to combine color information for depth inpainting. This color-similarity term is based on an assumption that neighboring pixels with similar color are likely to have similar depth values. In addition, there are filter terms which decide that the contribution of depth values to an unknown pixel varies according to geometric distance and direction. Additionally, the pixels near the edges of the color image are estimated later than the pixels which are far away from them to preserve fine edges. Overall, the interface of enhanced photography algorithms is hardware accelerated with OpenCL, so it is computationally efficient to be used in the approach to obtain smoother and denser depth images, which are beneficial for both the detection and the expansion of the traversable area. Shown in Figure 5, the presented approach remarkably smooths and improves the density of the original depth image from the RealSense sensor: firstly, the horizontal field angle of depth image has increased from 59° to 70°, which is the field angle of the color camera, allowing for broader detection; secondly, the filtered depth image has far less noise and fewer mismatches than the original depth image; lastly, the guided filtered depth image achieves 100% density.

### 3.2. Preliminary Ground Segmentation

In order to detect the ground, a simple and effective technique is presented. Firstly, 3D coordinates of the point cloud are calculated. Given the depth Z of pixel (u,v) in the depth image, the calibrated focal length f, and $\left(u_{0},v_{0}\right)$ the principal point, the point cloud in the camera coordinate system can be determined using Equations (1) and (2):

(1) $$X=Z\times \frac{u-u_{0}}{f}$$

(2) $$Y=Z\times \frac{v-v_{0}}{f}$$

On the strength of the attitude sensor, X, Y, and Z coordinates in the camera coordinate system can be adjusted to world coordinates. Assume a point in the camera coordinate system is (X,Y,Z) and the attitude angles acquired from the attitude sensor are (a,b,c). This means the point (X,Y,Z) rotates about the *x*-axis by α=a, then rotates about the *y*-axis by β=b and rotates about *z*-axis by γ=c in the end. Shown in Equation (3), multiplying the point (X,Y,Z) by the rotation matrix, and the point $\left(X_{w},Y_{w},Z_{w}\right)$ in world coordinates is obtained:

(3) $$\left[\begin{array}{l} X_{w}\\ Y_{w}\\ Z_{w} \end{array}\right]=\left[\begin{array}{ccc} \cos\gamma & -\sin\gamma & 0\\ \sin\gamma & \cos\gamma & 0\\ 0 & 0 & 1 \end{array}\right]\left[\begin{array}{ccc} \cos\beta & 0 & \sin\beta \\ 0 & 1 & 0\\ -\sin\beta & 0 & \cos\beta \end{array}\right]\left[\begin{array}{ccc} 1 & 0 & 0\\ 0 & \cos\alpha & -\sin\alpha \\ 0 & \sin\alpha & \cos\alpha \end{array}\right]\left[\begin{array}{l} X\\ Y\\ Z \end{array}\right]$$

The ground plane detection is based on the RANdom SAmple Consensus (RANSAC) algorithm [32]. By using the plane model, the RANSAC algorithm provides a robust estimation of the dominant plane parameters, performing a random search to detect short-range ground preliminarily, which is assumed to be the largest plane in the scenario. Although the assumption is violated in some real-world scenes, attitude angles of the camera and real vertical heights are employed to restrict the sampling process. The plane model is shown in Equation (4), and the inlier points of ground are determined with Equation (5). Firstly, a set of 3D points are randomly chosen from the point cloud to solve for the initial parameters A, B, C, and D. Secondly, the remaining 3D points are validated to count the number of inliers. After m computations, the ground plane is determined, which is the plane with the most inlier points. For the RANSAC algorithm, shown in Equation (6), if P is the probability of not failing the computation of outliers, p is the dimension of the model (three in our case), and η is the overall percentage of outliers, the number of computed solutions m can be selected to avoid overall sampling error:

(4) $$AX_{w}+BY_{w}+CZ_{w}+D=0$$

(5) $$d(X_{w},Y_{w},Z_{w})=\frac{\left|AX_{w}+BY_{w}+CZ_{w}+D\right|}{\sqrt{A^{2}+B^{2}+C^{2}}}<T$$

(6) $$m=\frac{log(1-P)}{log(1-{\left(1-\eta \right)}^{p})}$$

Rather than generate ground plane segmentation with the original point cloud, points are adjusted from the camera coordinate system to the world coordinate system in consideration of three respects:

- The inclination angle θ of the sampled plane can be calculated using Equation (7). This allows for dismissing some sample errors described in [25]. For example, if inclination angle of a sampled plane is abnormally high, the plane could not be the ground plane.
- Since the incorrect sampled planes are dismissed directly, the validation of inlier 3D points can be skipped to save much computing time.
- Given points in the world coordinate system, we obtain a subset of 3D points which only contains points whose real height is reasonable to be ground according to the position of the camera while the prototype is worn. Points which could not be ground points, such as points in the upper air are not included. As a result, η the percentage of outliers is decreased, so m, the number of computations, is decreased and, thereby, a great deal of processing time is saved.

(7) $$\theta =arccos\frac{\left|B\right|}{\sqrt{A^{2}+B^{2}+C^{2}}}$$

**Figure 6:**
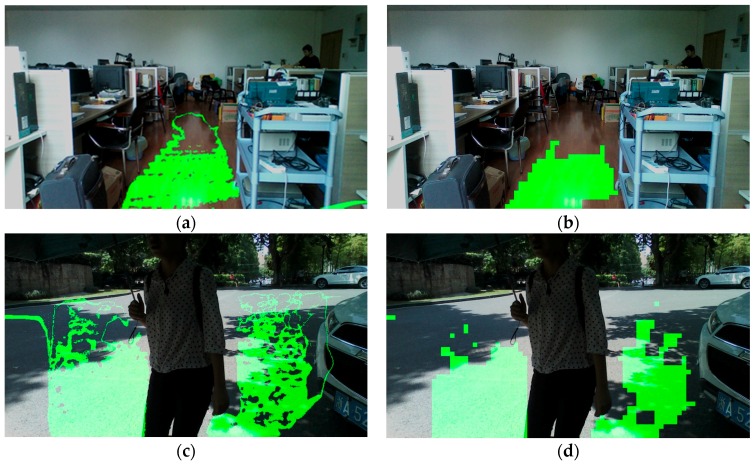
Ground plane segmentation in indoor and outdoor environments. (**a**,**c**) Ground plane detection based on the RANSAC algorithm; (**b**,**d**) salient parts in the ground plane are dismissed with surface normal vector estimation.

After initial ground segmentation, some salient parts, such as corners and little obstacles on the ground may be included in ground plane. Salient parts should be wiped out of the ground for two reasons: little obstacles may influence VIP; these parts may extend out of the ground area in the stage of seeded region growing. In this work, salient parts are removed from the ground based on surface normal vector estimation. Firstly, the depth image is separated into image patches; secondly, the surface normal vector of each patch is estimated through principal component analysis, the details of which are described in [14]; lastly, patches whose normal vector has a low component in the vertical direction are discarded. In the sampling stage, the number of iterations m equals 25, and inclination angle threshold of the ground plane is empirically set to 10°. Figure 6 depicts examples of short-range ground plane segmentation in indoor and outdoor environments, both of them achieving good performance, detecting the ground plane and dismissing salient parts correctly.

### 3.3. Seeded Region Growing

In order to expand traversable area to longer and broader range, a seeded region growing algorithm is proposed, combining both color images and filtered depth images. Instead of attaching importance to thresholds, edges of the color image are also adequately considered to restrict growth to other obstacle regions.

Firstly, seeds are chosen according to preliminary ground detection. A pixel is set as a seed to grow if two conditions are satisfied: the pixel is within the ground plane; four-connected neighbor pixels are not all within the ground plane. The seeds are pushed into the stack.

Secondly, a seed is valid to grow when it meets two conditions: the seed has not been traversed before, which means each seed will be processed only once; the seed does not belong to the edges of the color image.

Thirdly, we assume the growing starts from pixel G, whose depth value is d and hue value is v. One of the four-connected neighbors is Gi, whose depth value is $d_{i}$ and hue value is $v_{i}$. Whether Gi belongs to G’s region and be classified as traversable area depends on the following four growing conditions:

- Gi is not located at Canny edges of color image;
- Gi has not been traversed during the expansion stage;
- Real height of Gi is reasonable to be included in traversable area; and
- $\left|v-v_{i}\right|<\delta_{1}$ or $\begin{array}{l} \left|v-v_{i}\right|<\delta_{2}\\ \left|d-d_{i}\right|<\delta_{h} \end{array}$, where $\delta_{1}$ is the lower hue growing threshold, and $\delta_{2}$ is the higher growing threshold, while $\delta_{h}$ the height growing threshold, limits the expansion with only the color image.

**Figure 7:**
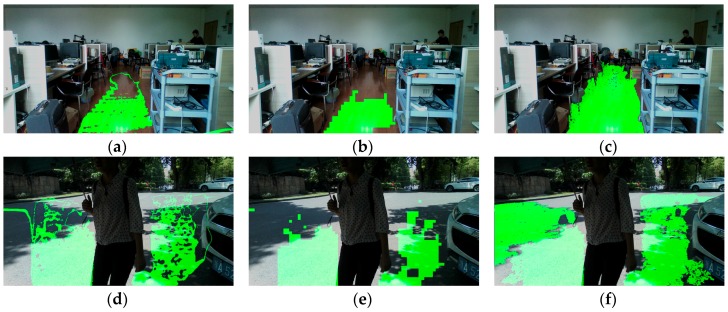
Traversable area expansion in indoor and outdoor environments. (**a**,**d**) Ground plane detection based on the RANSAC algorithm; (**b**,**e**) salient parts in the ground plane are dismissed with surface normal vector estimation; and (**c**,**f**) preliminary traversable area are expanded greatly with seeded region growing.

If all four conditions are true, Gi is qualified for the region grown from G, so Gi is classified as a traversable area. Each qualified neighbor pixel is put into the stack. When all of G’s four-connected pixels have been traversed, pop G out of the stack and let Gi be the new seed and repeat the above process. When the stack is empty, the seeded growing course finishes. After the seeded growing stage, the short-range ground plane has been enlarged to a longer and broader traversable area. Figure 7 depicts examples of expansion based on seeded region growing under indoor and outdoor situations, both expanding the traversable area to a great extent and preventing growth into other non-ground areas.

## 4. Experiment

In this section, experimental results are presented to validate our approach for traversable area detection. The approach is tested on a score of indoor and outdoor scenarios including offices, corridors, roads, playgrounds, and so on.

**Figure 8:**
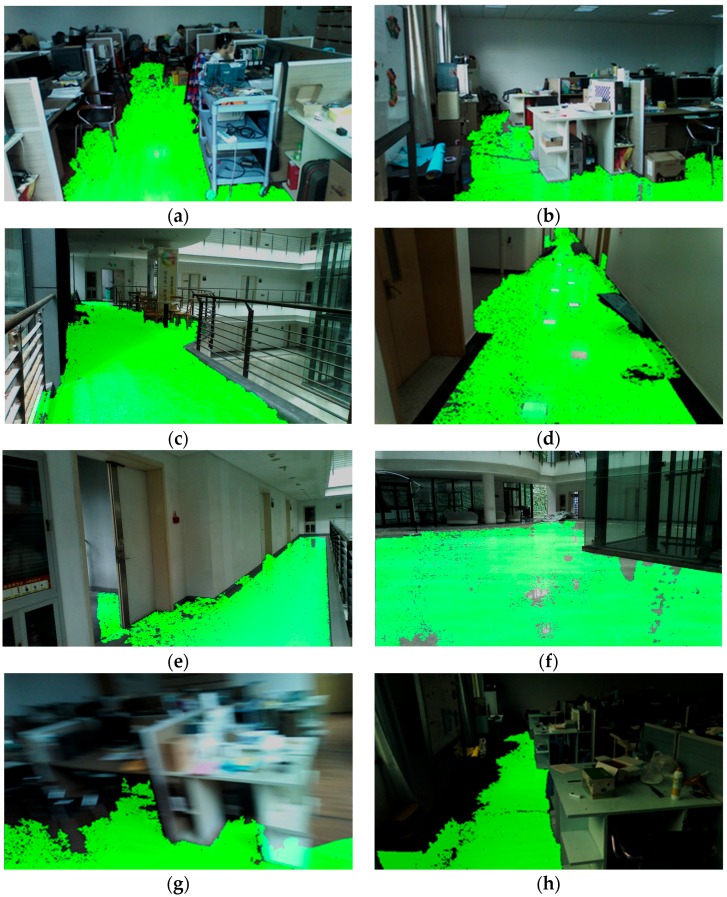
Results of traversable area expansion in indoor environment. (**a**,**b**) Traversable area detection in offices; (**c**–**e**) traversable detection in corridors; (**f**) traversable area detection in an open area; (**g**) traversable area detection with color image blurring; abd (**h**) traversable area detection with color image under-exposing.

Figure 8 shows a number of traversable area detection results in the indoor environment. Largely-expanded traversable area provides two superiorities: firstly, longer range allows high-level path planning in advance; and, secondly, broader range allows precognition of various bends and corners. For special situations, such as color image blurring and image under-exposing, the approach still detects and expands the traversable area correctly, as shown in Figure 8g,h. Additionally, the approach is robust regardless of continuous movement of the cameras as the user wanders in real-world scenes. 

**Figure 9:**
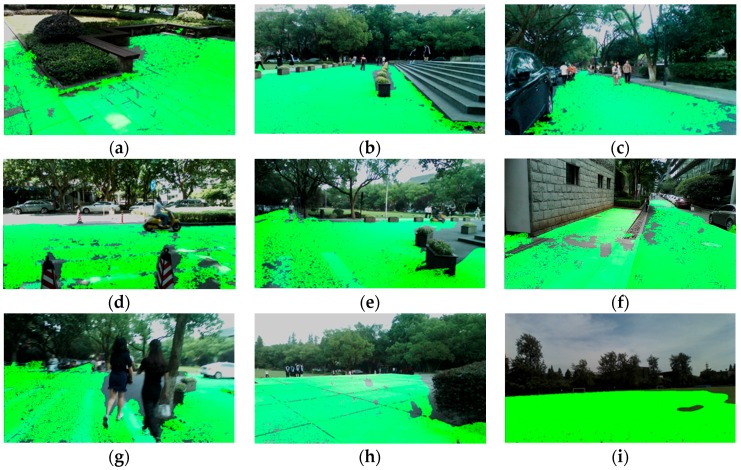
Results of traversable area expansion in outdoor environment. (**a**–**g**) Traversable area detection on roads; (**h**) traversable area detection on a platform; and (**i**) traversable area detection on a playground.

Figure 9 shows several traversable area detection results under outdoor circumstances. It can be seen that traversable area has been enlarged greatly out to the horizon. Rather than the short-range ground plane, the expanded traversable area frees the VIP to wander in the environment.

**Figure 10:**
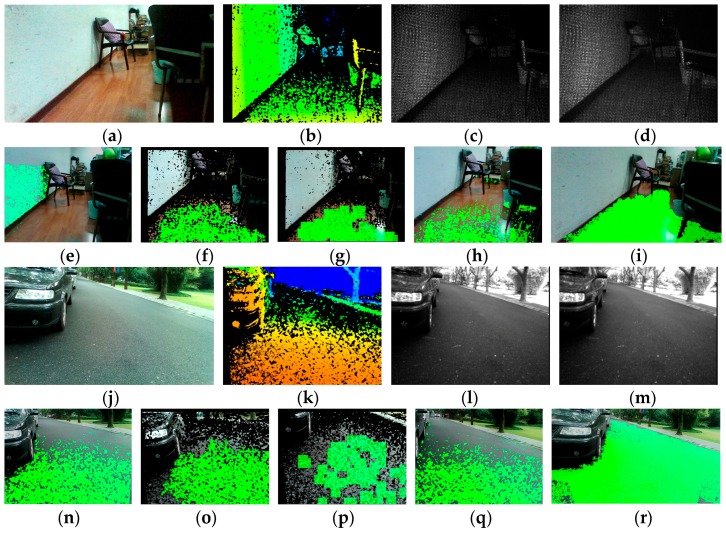
Comparisons of results of different traversable area detection approaches. (**a**–**d**) The set of images of a typical indoor scenario including color image, depth map, and calibrated IR pairs; (**e**–**i**) the results of different approaches on the indoor scenario; (**j**–**m**) the set of images of a typical outdoor scenario; and (**n**–**r**) the results of different approaches on the outdoor scenario.

To compare the performance of traversable area detection with respect to other works in the literature, the results of several traversable detection approaches on a typical indoor scenario and outdoor scenario are shown in Figure 10. Given the depth image, the approach proposed by Rodríguez estimated the ground plane based on RANSAC plus filtering techniques [25]. Figure 10n is a correct result of detecting the local ground, but the wall is wrongly detected as the ground plane in Figure 10e, which is one type of sample error mentioned in the paper. This kind of error is dissolvable in our work with consideration of the inclination angle of the plane. The approach proposed by Cheng detected the ground with seeded region growing of depth information [15]. The approach in [15] projects RGB information onto the valid pixels of depth map, so the detecting result shown in Figure 10f,o has many noises and black holes, and the detecting range is restricted since the depth information is discrete and prone to inaccuracy in long range. However, the main problem of the algorithm lies in that the seed pixels are elected randomly, thereby causes intolerable fluctuations to confuse VIP. In our previous works, we only employed depth information delivered by the light-coding sensor of the Microsoft Kinect [14,15]. However, the sensor outputs a dense 3D point cloud (ranges from 0.8 m to 5 m) indoors and fails in sunny outdoor environments. As a result, the algorithms are unable to perform well when the sensor could not generate a dense map. In Figure 10g,p, the idea of using surface normal vectors to segment ground presented in [14,40] is able to segment the local ground plane but fails to segment the long-range traversable area robustly as the estimation of normal vectors asks the sensor to produce dense and accurate point clouds. In this paper, we fully combine RGB information and depth information to expand the local ground plane segmentation to long range. In the process, IR image large-scale matching and RGB image guided filtering are incorporated to enhance the depth images. Although the computing time improves from 280 ms to 610 ms per frame on a 1.90 GHz Intel Core Processor, within which the RGB image-guided filtering is hardware accelerated with the HD4400 integrated graphics, the range of traversable detection has been expanded to a great extent and the computing time contributed in this process endows VIPs to perceive traversability at long range and plan routes in advance so the traversing time eventually declines. Figure 10h,q shows the results of traversable area detection without IR image large-scale matching and RGB image-guided filtering. The seeded region growing process is unable to enlarge the local ground segmentation based on RANSAC to long-range as the depth map is still discrete and sparse in the distance. Comparatively, in Figure 10i,r, after IR image large-scale matching and RGB image-guided filtering, the segmented local ground plane largely grows to a longer and broader traversable area. The set of our images is available online at Kaiwei Wang Team [52].

**Figure 11:**
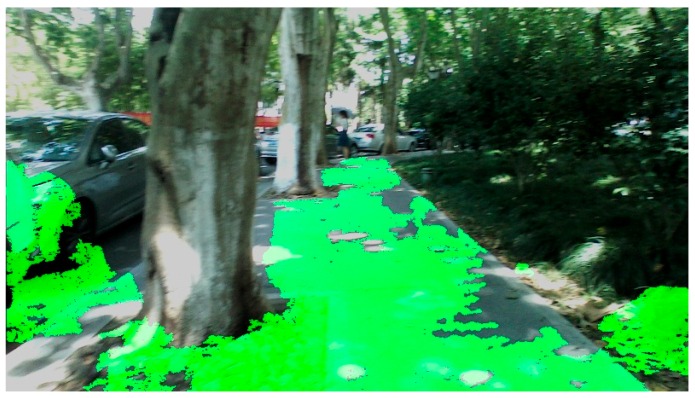
An example of expansion error. The ground has been unexpectedly expanded to a part of the car.

The approach creates a multithreaded program including a thread for image acquisition and depth enhancement, a thread for traversable area detection and expansion, as well as a thread for audio interface generation for the VIP. Together, the average processing time of a single frame is 610 ms on a 1.90 GHz Intel Core 5 processor, making the refresh rate of the VIP audio feedback 1.6 times per second. In addition, detection rate and expansion error for indoor and outdoor scenarios are presented to demonstrate the robustness and reliability of the approach. Indoor scenarios, including a complicated office room and a corridor are analyzed, while outdoor scenarios, including school roads and a playground, are evaluated. Typical results of the four scenarios are depicted in Figure 8a,c and Figure 9c,i. As depicted in Figure 11, part of the car has been classified as traversable area, which is a typical example of expansion error.

In order to provide a quantitative evaluation of the approach, given Equations (8) and (9), detection rate (*DR*) is defined as the number of frames which ground has been detected correctly (*GD*) divided by the number of frames with ground (*G*). Meanwhile, expansion error (*EE*) is defined as the number of frames which traversable area has been expanded to non-ground areas (*ENG*) divided by the number of frames with ground (*G*):

(8) DR=GD/G

(9) EE=ENG/G

Shown in Table 1, detection rates of the four scenarios are all above 90%, demonstrating the robustness of the approach. For the scene of the corridor, it yields an expansion error of 15.9%. This is mainly due to inadequate lighting on the corners in the corridor, so the edges of the color image are fuzzy and the traversable area may be grown to the wall. Overall, the average expansion error is 7.8%, illustrating the reliability of the approach, which seldom recognizes hazardous obstacles as safe traversable area.

Additionally, the average density of depth images of four different scenarios is calculated to prove that IR image large-scale matching and RGB image guided filtering remarkably improve the density of the original depth image from the RealSense sensor. The density of the depth image is defined as the number of valid pixels divided by the resolution. As shown in Table 2, the average density of the large-scale matched depth image is much higher than the original depth image and the guided-filtered depth image achieves 100% density.

## 5. User Study

In this section, a user study is elaborated in terms of assisting system overview, non-semantic stereophonic interface, and assisting performance study.

### 5.1. Assisting System Overview

**Figure 12:**
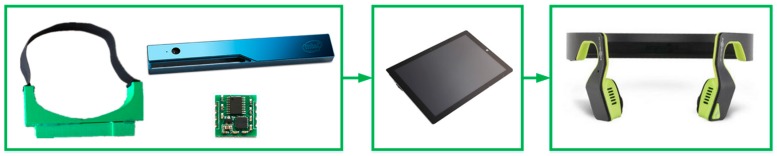
The assisting system consists a frame which holds the RealSense R200 and the attitude sensor, a processor, and a bone-conducting headphone.

The approach presented has been integrated in an assisting system. As shown in Figure 12, the system is composed of a RGB-D RealSense R200sensor, an attitude sensor MPU6050, a 3D-printed frame which holds the sensors, a processor Microsoft Surface Pro 3, as well as a bone-conducting headphone AfterShokz BLUEZ 2S [53], which transfers non-semantic stereophonic feedback to the VIP. Since the RealSense R200 only uses part of the USB 3.0 interface to transmit data, spare interfaces which are compatible with USB 2.0 are employed to transmit attitude angles from the MPU6050. Additionally, the processor communicates with the headphone through Bluetooth 4.0. Thereby, the system only needs a USB 3.0 cord to transfer images and data from sensors to the processor. As we know, VIP rely on voices from the environment great deal. For example, they use the sounds from cars to understand the orientation of streets. The assisting prototype is not only wearable but also ears-free, because the bone-conducting interface will not block VIP’s ears from hearing environmental sounds.

### 5.2. Non-Semantic Stereophonic Interface

The assisting system uses a non-semantic stereophonic interface to transfer traversable area detection results to the VIP. The generation of the non-semantic stereophonic interface follows rules below:

- Divide the detection result into five directions, since the horizontal field view has been enlarged from 59° to 70°, so each direction corresponds to traversable area with a range of 14°.
- Each direction of traversable area is represented by a musical instrument in 3D space.
- In each direction, the longer the traversable area, the greater the sound from the instrument.
- In each direction, the wider the traversable area, the higher the pitch of the instrument.

**Figure 13:**
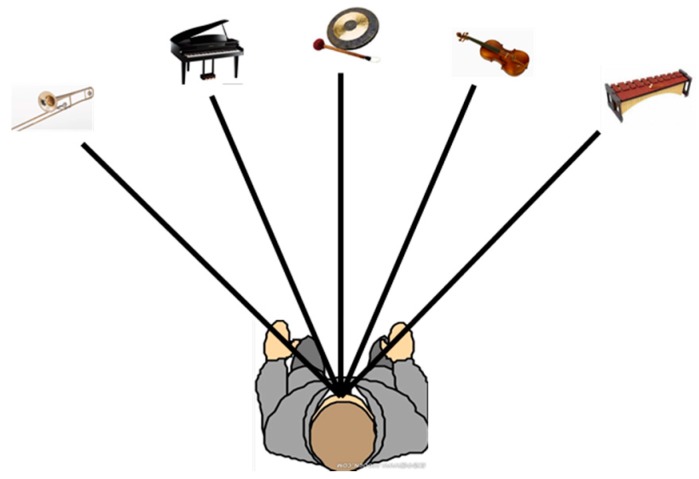
Non-semantic stereophonic interface of the assisting system. Sounds of five directions of traversable area are presented by five musical instruments in 3D space, including trumpet, piano, gong, violin, and xylophone.

To sum up, the directions of traversable area are differentiated not only by sound source locations in 3D space, but also by musical instruments, whose tone differs from each other. As shown in Figure 13, five instruments, including trumpet, piano, gong, violin, and xylophone, produce sounds simultaneously which last for 0.6 s, notifying the user the traversable area. Additionally, we also implemented a simple obstacle detection method to warn against walking on the ground under obstacles in the air (e.g., Figure 8g). The 3D points which are not within traversable area and are within close range (1 m in our case) are counted in respectively five directions. If the number of points in one direction exceeds a threshold, it means there is one obstacle in the close range. In this case, the audio interface generates a friendly prompt to help VIP to be aware of close obstacles. Since that is not the major topic of this paper, specific parameters of the audio feedback are not discussed here.

### 5.3. Assisting Performance Study

**Figure 14:**
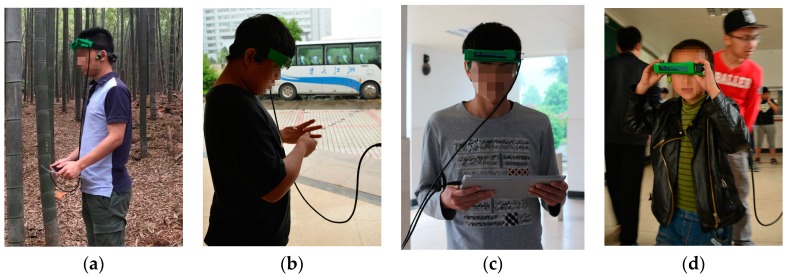
Eight visually impaired volunteers took part (**a**–**d**). The moments of the assisting study. Participants’ faces are blurred for the protection of the privacy (we have gotten the approval to use the assisting performance study for research work).

Eight visually impaired volunteers including three suffering from total blindness participated in the user study. Figure 14 are the moments of the assisting study. During the assisting study, participants first learned the audio feedback. The working pattern of the system and signals from the headphone was introduced. Each one of them has ten minutes to learn, adapt to the audio interface, and wander around casually. After that, participants were asked to traverse through obstacles without collisions, and finally find the person standing at the end point. A contrary test is designed to compare its performance under two conditions: the signal from the audio interface is generated according to the original ground detection, and the audio interface is generated according to the traversable area expansion.

**Figure 15:**
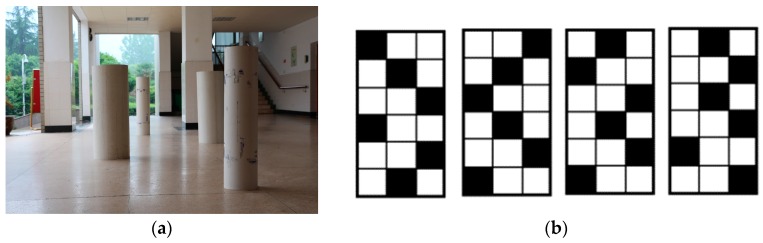
Obstacle arrangements. (**a**) An image of obstacle arrangement; (**b**) Four other obstacle arrangements.

After the learning stage, eight visually impaired participants were required to travel through obstacles. Shown in Figure 15, six different white boards including large columns were employed as obstacles. Five different obstacle arrangements were generated by arranging the position of obstacles differently. Firstly, they were asked to complete the course with traversable area expansion, and a typical detection example is shown in Figure 7. Secondly, they were asked to complete the course with original ground detection, which is shown in Figure 2. All visually impaired participants completed the test and found the person standing at the end point. The average number of collisions, average time and average number of steps to complete a single test were recorded. Collisions include collisions with obstacles and walls. The timer starts when a participant is sent to the start region and stops when the participant completes a single test. The distance between the start region and the end point is the same for all tests. However, the number of steps to complete a single test varies. As shown in Table 3, average number of collisions to complete a single test with traversable area expansion is 78.6% less than that with original ground detection. Most of the collisions occurred when the user did not know which direction to walk as the original ground detection is at short-range. Additionally, the average time to complete a single test with traversable area expansion is 29.5% less than that with original ground detection. Moreover, the number of average steps to complete a single test with traversable area expansion is 43.4% less than that with original ground detection. It is the expansion of traversable area which endows VIP the ability to plot routes farther ahead and, therefore, reduce traversing time and the number of steps. Each participant completes the tests with different obstacle arrangements in a random order. As a result, the participants have no idea about the arrangement of obstacles each time. It is ruled out that the decrease of collisions and traversing time after traversable area expansion is due to variation of familiarity with the prototype. Since the test was taken with traversable area expansion first and the taken with original ground detection afterwards, if it was due to the variation of familiarity, it would enhance rather than weaken the performance of navigational assistance, such as the number of collisions and the traversing time taken with traversable expansion would be more than with original ground detection. It can be proved convincingly that traversable area expansion improves the performance dramatically. In other word, the safety and robustness enhances navigation.

After the test, eight participants were asked two simple questions including whether the prototype is easy to wear and whether the system provides convenient assistance to travel in an unfamiliar environment. Shown in the questionnaire (Table 4), all users answered that the system is useful and can offer help in unknown or intricate environments. It not only gave us significant confidence, but also demonstrated usefulness and reliability of the approach. In addition, some users gave some advice on adding functions, such as face recognition or GPS navigation and a user hopes that the prototype be designed in a hat.

## 6. Conclusions

RGB-D sensors are a ubiquitous choice to provide navigational assistance for visually impaired people, with good portability, functional diversity, and cost-effectiveness. However, most assisting solutions, such as traversable area awareness, suffer from the limitations imposed by RGB-D sensor ranging, which is short, narrow, and prone to failure. In this paper, an effective approach is proposed to expand ground detection results to a longer and broader range with a commercial RGB-D sensor, the Intel RealSense R200, which is compatible with both indoor and outdoor environments. Firstly, the depth image of the RealSense is enhanced with large scale matching and color guided filtering. Secondly, preliminary ground segmentation is obtained by the RANSAC algorithm. The segmentation is combined with an attitude sensor, which eliminates many sample errors and improves the robustness of the preliminary result. Lastly, the preliminary ground detection is expanded with seeded region growing, which fully combines depth, attitude, and color information. The horizontal field angle of the traversable area has been increased from 59° to 70°. Additionally, the expansion endows VIP the ability to predict traversability and plan paths in advance since the range has been enlarged greatly to a large extent. The approach is able to see smoothly to the horizon, being acutely aware of the traversable area at distances far beyond 10 m. Both indoor and outdoor empirical evidences are provided to demonstrate the robustness of the approach, in terms of image processing results, detection rate, and expansion error. In addition, a user study is described in detail, which proves the approach to be usable and reliable.

In the future, we aim to incessantly enhance our navigational assistance approach for the visually impaired. Especially, the implementation of the algorithm is not yet optimized, so we are looking forward to speeding it up. Additionally, a cross-modal stereo-matching scheme between IR images and RGB images would also be interesting and useful to inherently improve the detecting range and ranging accuracy of the camera.

## Figures and Tables

**Table 1:** Detection rate and expansion error of the approach.

Scenario	Frames with Ground (*G*)	FRAMES Detected Ground Correctly (*GD*)	Detection Rate (*DR*)	Frames Expanded to Non-Ground Areas (*ENG*)	Expansion Error (*EE*)
An office	1361	1259	92.5%	44	3.2%
A corridor	633	614	97.0%	101	15.9%
School roads	837	797	95.2%	81	9.7%
A playground	231	228	98.7%	13	5.6%
All	3062	2898	94.4%	239	7.8%

**Table 2:** Average density of depth images including the original depth image, large-scale matched depth image and guided-filtered depth image.

Scenario	Original Depth Image (Resolution: 293,904)	Large Scale Mathced Depth Image (Resolution: 293,904)	Guided Filtered Depth Image (Resolution: 360,000)
An office	68.6%	89.4%	100%
A corridor	61.4%	84.5%	100%
School roads	76.2%	91.2%	100%
A playground	79.5%	92.0%	100%

**Table 3:** Number of collisions and time to complete tests in two conditions: the audio interface transferred to the VIP is generated according to original ground detection or traversable area expansion.

Detection Result Transfered to VIP	Total Number of Collisions	Average Number of Collisions of Each Time	Total Time to Complete Tests	Average Time to Complete a Single Test	Total Number of Steps	Average Number of Steps to Complete a Single Test
Original ground deteciton	103	2.58	733 s	18.33 s	1850	46.25
Traversable area expansion	22	0.55	517 s	12.93 s	1047	26.18

**Table 4:** A questionnaire. After the test, eight participants were asked two simple questions.

User	Total Blind or Partially Sighted	Easy to Wear?	Useful?	Advice
User 1	Partially sighted	Yes	Yes	
User 2	Partially sighted	Yes	Yes	Add face recognition
User 3	Total blind	Yes	Yes	Design the prototype in a hat
User 4	Partially sighted	Yes	Yes	
User 5	Partially sighted	No	Yes	Add GPS navigation
User 6	Total blind	Yes	Yes	
User 7	Total blind	No	Yes	
User 8	Partially sighted	Yes	Yes	
